# Cases Admitted under the Care of the Surgeon of the Finsbury Dispensary, St. John's Square, Clerkenwell, from October 10, to November 10, 1802

**Published:** 1802-12-01

**Authors:** J. Ricards

**Affiliations:** 68, Hatton Street


*Cases admitted under the Care of the Surgeon of the Finsbury Dispensary, St. John's Square, Clerkenwell, from October* 10, *to November* 10, 1802.

| | | | |
|---|---|---|---|
| Phlegmone Teftis | 1 | Contufiones | 2 |
| Maftodynia | 3 | Lues | 3 |
| Paronychia | 3 | Gonorrhœa Impura | 2 |
| Pernio | 1 | ——— Mucofa | 1 |
| Sarulis | 1 | Vacciola | 6 |
| Abfceffus Genu | 1 | Schirrus Mammæ | 1 |
| ——— Maxilla | 1 | Hydarthrus Genu† | 1 |
| ——— Scrophulofi | 7 | ——— Ifchû | 1 |
| Sphacelus Faciei | 1 | Hydrocele | 1 |
| ——— Dorfi | 1 | Hernia | 1 |
| Ulcera Artuum | 7 | Spina Incurvata | 1 |
| Rhagas Mamillæ | 1 | Prolapfus Ani | 1 |
| Fiftula in Perinæo | 1 | ——— Uteri | 1 |
| Leucoma | 1 | Dyfecæa | 1 |
| Combufturæ | 4 | Varix | 1 |
| Fracturæ Coftarum | 2 | Tinea | 1 |
| Spafma Cubiti* | 1 | Eruptiones Chronicæ | 3 |
| Paralyfis Traumatica | 1 | | |
| Vulnus Artus | 1 | Total | 67 |

---

* The injury which we ufually denominate, in common language, a fprain, does not feem to have been diftinguifhed as of a peculiar nature, by any Nofologift, except Vogel, who intends, if I underftand him right, to exprefs this accident by the term *spasma*, Gen. 479, "*Species solutionis continui tendinum*, vel *ligamentorum* CITRA RUPTURAM, *membri mobilitatem dolorificam inducens*." Other Nofologifts do not make any diftinction between fprain and rupture, as under the genus Ruptura, both varieties of the accident feem to be defined; thus Sauvage defines Ruptura, "*Tendinum, &c. &c. Solutio*, vel VEHEMENS DISTRACTIO;" while Vogel, as above, feems to diftinguifh them, as appears to me with propriety, "*Citra Rupturam*." For although in fprains fome fibres may be in many inftances torn afunder, yet I fhould prefer applying the word Ruptura to thofe accidents which are evidently attended with rupture of tendons, &c. and Spafma to thofe which are not evidently fo connected, and which we ufually term Sprains. In the nofological arrangement of chirurgical difeafes, by Mr. H. Monro, the author has claffed this accident in the fame fpecies with "Contufio."

† Cullen has called the difeafe, White Swelling, Hydarthrus; which however, does not appear to exprefs the real nature of the difeafe, as Hydarthrus would feem to imply a dropfical accumulation in a joint, which is not the cafe in

The cafe which I have denominated Paralyfis Traumatica, happened in a young woman, nineteen years of age. About the begining of October, fhe cut the extremity of the little finger nearly off; which wound, however, produced at firft no uncommon fymptoms, and healed very readily; a fortnight after the accident, the pain in the finger increafed, extended along the arm, and an abforbent gland at the elbow became enlarged and very painful; about a week from the commencement of this enlargement, fhe found fuddenly, that fhe was deprived of fenfation in the whole arm, from the fhoulder downward, and likewife the power of voluntary motion.

A week after this time (Nov. 3) fhe became a patient at the Difpenfary; at which time the paralyfis of the arm continued very complete, there being a perfect abolition of both fenfation and voluntary motion, except that when fome powerful electric fhocks were paffed through the arm, they were flightly felt; her pulfe was equal in both wrifts, and of a natural degree of fullnefs and velocity; the paralytic arm was colder than the other; fhe complained of fome pain in the head, and her bowels were conftipated.

After paffing fome electrical fhocks through the arm, and likewife fubjecting it to the electric friction, I directed a blifter to the fore part of the arm, and that the remaining furface fhould be rubbed with muftard, with a cathartic internally.

Nov. 6, No alteration with refpect to the paralyfis, but the pain in the head increafed. Applic. emplaftrum canthari[illegible]. ad nuch. Calomel, gr. ij. omni nocte. Hauft. cath. omni mane. Continue the electricity and friction.

Nov. 8, A flight degree of fenfation in the arm, but no voluntary power; directed the calomel every night, and the cathartic medicine every morning, to be continued, and that fhe fhould take, likewife, twice a day, tinct. guaiac. ammon. ℥fs.

Nov. 10, This morning the perfect fenfation and power of voluntary motion returned as fuddenly as they had left her; the enlargement of the lymphatic gland has difappeared feveral days; there now remains an aching pain in the arm. I recommended a continuance of ʒij. of tinct. guaiac. bis die.

Whether this very fudden attack could be attributed in any meafure to the wound of the finger I very much doubt; as it is a circumftance not eafy to be accounted for, how an injury, or divifion

---

in the true White Swelling; I have however retained the name from his authority, and have given the fame appellation (Hydarthrus Ifchii) to that difeafe of the hip which is ufually known by the very indefinite term the *Hip-Cafe*, as it appears to be of the fame nature as the Scrophulous White Swelling of the Knee.

division of the extremity of a remote branch of a nerve could deprive the trunk of that nerve of its natural functions; in fact, not one trunk alone, but the whole of the nerves emanating from the axillary plexus. We have heard of locked jaw, and other convulsive motions produced by slight wounds of the extremities, but never, as I recollect, of a complete paralysis; and although there was inflammation of an absorbent, as was evident from the swelling of the lymphatic gland, yet I think the paralysis of the arm must be attributed to other causes.

A case of tumour of the throat was admitted a few weeks since, which was attended by some peculiar circumstances; the disease existed in the person of a young woman, and had every appearance of bronchocele, being a swelling of a diffused nature, rather soft, and colourless; had appeared very gradually, and occupied the situation of the thyroid gland, confining itself principally to the right side; it was unattended by pain, but was so large as to cause considerable uneasiness and difficulty in swallowing from the pressure communicated to the œsophagus. I at first directed the free internal administration of spong. ust. and applied externally cloths kept constantly moist in a solution of ammon. mur. in vinegar. Under this treatment for several weeks, no alteration with respect to the size of the tumour had occurred. After this time I perceived that one particular part became more prominent than the rest; not in the centre of the tumour, but nearly at the circumference, and over the centre of the throat. This portion continued to project till it had formed a kind of mamillary process, very different from the usual regular elevation of an abscess. This projection soon became inflamed, and after a few days appeared to contain matter, when I opened it, and discharged some, which did not much reduce the size of the principal swelling. The opening, after the first day, discharged much serous fluid, nearly limpid, but very little pus, and gradually the tumour began to subside. As the wound seemed to have great propensity to heal, I introduced a pea daily, to promote and accelerate the discharge; after the continuance of which treatment about a fortnight, as the tumour had totally subsided, I suffered the small aperture to close.

This case appears to me to point out the good effects which are likely to ensue from the establishment of a drain in cases of Bronchocele, as by the passing of a seton through the gland, which offers a very reasonable probability of success; this has been performed in several cases, but with what result I am not acquainted. The disease is one of those which frequently continue uncured from the great length of time generally necessary to produce any change; the spong. ust. has often been found very successful, and would no doubt prove so much more frequently quently, but that perſons have ſeldom patience and reſolution to continue its uſe for a ſufficient length of time. Any mean, therefore, which would afford a cure in a ſhort ſpace of time, comparatively, would be particularly valuable, eſpecially in thoſe countries where the diſeaſe is endemial.

68, *Hatton Street.* J. RICARDS.

